# Generalized Quantum Convolution for Multidimensional Data

**DOI:** 10.3390/e25111503

**Published:** 2023-10-31

**Authors:** Mingyoung Jeng, Alvir Nobel, Vinayak Jha, David Levy, Dylan Kneidel, Manu Chaudhary, Ishraq Islam, Muhammad Momin Rahman, Esam El-Araby

**Affiliations:** Department of Electrical Engineering and Computer Science, University of Kansas, Lawrence, KS 66045, USA; islam.alvir@ku.edu (A.N.); vinayakjha@ku.edu (V.J.); david.levy@ku.edu (D.L.); dckneidel@ku.edu (D.K.); manu.chaudhary@ku.edu (M.C.); ishraq@ku.edu (I.I.); momin.rahman@ku.edu (M.M.R.); esam@ku.edu (E.E.-A.)

**Keywords:** convolution, quantum algorithms, quantum image processing, quantum computing

## Abstract

The convolution operation plays a vital role in a wide range of critical algorithms across various domains, such as digital image processing, convolutional neural networks, and quantum machine learning. In existing implementations, particularly in quantum neural networks, convolution operations are usually approximated by the application of filters with data strides that are equal to the filter window sizes. One challenge with these implementations is preserving the spatial and temporal localities of the input features, specifically for data with higher dimensions. In addition, the deep circuits required to perform quantum convolution with a unity stride, especially for multidimensional data, increase the risk of violating decoherence constraints. In this work, we propose depth-optimized circuits for performing generalized multidimensional quantum convolution operations with unity stride targeting applications that process data with high dimensions, such as hyperspectral imagery and remote sensing. We experimentally evaluate and demonstrate the applicability of the proposed techniques by using real-world, high-resolution, multidimensional image data on a state-of-the-art quantum simulator from IBM Quantum.

## 1. Introduction

Convolution is a common operation that is leveraged in a wide variety of practical applications, such as signal processing [1], image processing [2], and most recently, convolutional neural networks [3]. However, leveraging the widespread utility of convolution operations in quantum algorithms is limited by the lack of a systematic, generalized implementation of quantum convolution. Specifically, contemporary quantum circuits for performing quantum convolution with a given filter are designed on a case-by-case basis [4,5,6,7,8]. In other words, implementing a novel convolution filter on a quantum computer is arduous and time consuming, requiring substantial human effort. Such a workflow is impractical for applications, such as quantum convolutional neural networks, which require a generalized, parameterized quantum circuit to iteratively test thousands of unique filters per training cycle.

In this work, we propose a generalizable algorithm for quantum convolution compatible with amplitude-encoded multidimensional data that is able to implement arbitrary multidimensional filters. Furthermore, our proposed technique implements unity stride, which is essential for capturing the totality of local features in input data. We experimentally verify our technique by applying multiple filters on high-resolution, multidimensional images and report the fidelity of the quantum results against the classically computed expectations. The quantum circuits are implemented on a state-of-the-art quantum simulator from IBM Quantum [9] in both noise-free (as a statevector) and noisy environments. Compared to classical CPU- and GPU-based implementations of convolution, we achieve an exponential improvement in time complexity with respect to data size. Additionally, when compared to existing quantum implementations, we achieve improved circuit depth complexity when factoring in the data encoding.

The work is structured as follows. In Section 2, we cover important background information and review the related work. In Section 3, we introduce the proposed quantum convolution circuits and provide analyses of the corresponding circuit depth. In Section 4, we present the experimental setup and results, while in Section 5, we provide discussions of the results and comparisons to related work. Finally, in Section 6, we present our conclusions and potential avenues for future explorations and extensions.

## 2. Background

In this section, we discuss related work pertinent to quantum convolution. Quantum operations that are relevant to convolution, such as quantum data encoding, and quantum shift operation, are also presented.

### 2.1. Related Work

Classically, the convolution operation is implemented either directly or by leveraging *fast Fourier transform* (FFT). On CPUs, the direct implementation has a time complexity of $O(N^{2})$ [10], where *N* is the data size, while FFT-based implementation has a time complexity of O(NlogN) [10]. On GPUs, the FFT-based implementation has a similar O(NlogN) complexity [11]. It is also common to take advantage of the parallelism offered by GPUs to implement convolution using general matrix multiplications (GEMMs) with $O(N_{F}N)$ FLOPS [12,13], where $N_{F}$ is the filter size.

Techniques for performing quantum convolution have previously been reported [4,5,6,7,8]. However, these techniques only use fixed sizes of filter windows for specific filters, e.g., edge detection [4,5,6,7,8]. We will denote such methods as *fixed-filter* quantum convolution. Reportedly, these methods possess a quadratic circuit depth complexity of $O(n^{2})$ in terms of the number of qubits $n=\lceil \log_{2}N\rceil$, where *N* is the size of the input data [4,5,6,7,8]. Because the shortest execution time of classical convolution is in the order of O(N) or $O(2^{n})$ [12,13] with respect to data size *N*, authors of the quantum counterparts often claim a quantum advantage [4]. However, the reported depth complexity of *fixed-filter* quantum convolution does not include the unavoidable overhead of data encoding. Furthermore, there does not exist, to the best of our knowledge, a method for performing generalized, multidimensional quantum convolution.

In reported related work [4,5,6,7,8], data encoding is performed with either the *flexible representation of quantum images* (FRQI) [14] or *novel enhanced quantum representation* (NEQR) [15] methods. In these encoding techniques, positional information is stored in the basis quantum states of *n* qubits, while color information is stored via angle encoding and basis encoding for FRQI and NEQR, respectively. FRQI and NEQR require a total of n+1 and n+q qubits, respectively, where *q* is the number of qubits used to represent color values, e.g., q=8 for standard grayscale pixel representation. The reported circuit depth complexities of FRQI and NEQR are $O(4^{n})$ and $O(qn2^{n})$, respectively. When factoring in the depth complexities of either data-encoding technique, it is evident that the referenced *fixed-filter* quantum convolution techniques should be expected to perform worse than classical implementations.

In [16], the authors propose a method of edge detection based on amplitude encoding and the quantum wavelet transform (QWT), which they denote as *quantum Hadamard edge detection* (QHED). Although the work utilizes grayscale two-dimensional images, the QHED technique is highly customized for those data and does not easily scale or generalize to data of higher dimensions, such as colored and/or multispectral images. For example, the quantum discriminator operation in their technique is applied over all qubits in the circuit, without distinguishing between qubits representing each dimension, i.e., image rows or columns. Such a procedure not only forgoes parallelism and increases circuit depth but inhibits the algorithm’s ability to be generalized beyond capturing one-dimensional features.

In our proposed work, we achieve an exponential improvement in time complexity compared to classical implementations of convolution with respect to data size. Additionally, when compared to existing quantum convolution implementations, we achieve improved circuit depth complexity when factoring in the data encoding. The contribution of our work is analyzed, experimentally verified, and discussed in detail in Section 5.

### 2.2. Classical to Quantum (C2Q)

Our method of quantum convolution leverages *amplitude encoding*, which encodes *N* data values directly in the complex probability amplitudes $c_{i}\in C$ of the positional basis state |i〉 for an *n*-qubit state |ψ〉, where $n=\lceil \log_{2}N\rceil$ and 0≤i<n, see (1):

(1) $$|\psi \rangle =\sum_{i=0}^{2^{n}-1}c_{i}|i\rangle :c_{i}\in C.$$

**Figure 1:**
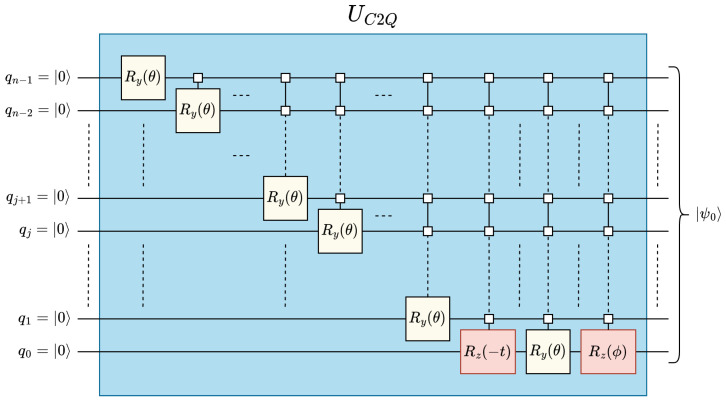
Quantum circuit for classical-to-quantum (C2Q) arbitrary state synthesis [17].

**Figure 2:**
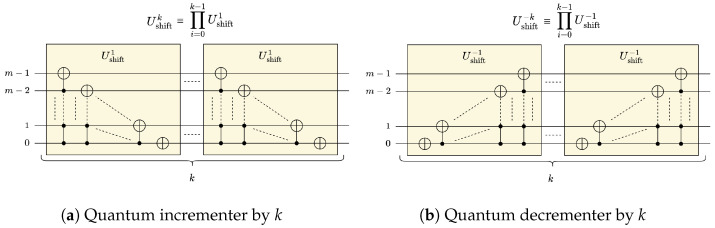
Quantum shift operation using quantum incrementers/decrementers.

We use the *classical-to-quantum* (C2Q) [17] data-encoding technique to encode the amplitude encoded state $|\psi_{0}\rangle$ from the ground state $|0\rangle^{⊗n}$, see Figure 1 and (2). The C2Q operation $U_{C2Q}$ has a circuit depth complexity of $O(2^{n})$, a quadratic and linear improvement over FRQI and NEQR, respectively:

(2) $$\begin{array}{cc} U_{C2Q}|0\rangle^{⊗n} & =|\psi_{0}\rangle \\ U_{C2Q} & =\left[\begin{array}{cc} |\psi_{0}\rangle & |\times \rangle \cdots |\times \rangle \end{array}\right],\;\mathrm{where}\\ |\times \rangle & =“\mathrm{don}’t\;\mathrm{care}” \end{array}$$

### 2.3. Quantum Shift Operation

A fundamental operation for quantum convolution is the *quantum shift operation*, denoted in this work as $U_{\mathrm{shift}}^{k}$, which shifts the basis states of the state vector by *k* positions when applied to an *m*-qubit state |ψ〉, see (3). The quantum shift operation is critical for performing the cyclic rotations needed to prepare strided windows when performing convolution. It is also common for the operation to be described as a *quantum incrementer* when k>0, see Figure 2a, and a *quantum decrementer* when k<0 [16,18], see Figure 2b:

(3) $$U_{\mathrm{shift}}^{k}\left|\psi \rangle =\sum_{i=0}^{2^{m}-1}c_{i}\right|j\rangle ,\;\mathrm{where}\;j=\left(i-k\right)\;\mathrm{mod}\;2^{m}$$

## 3. Materials and Methods

In general, a convolution operation can be performed using a sequence of shift and multiply-and-accumulate operations. In our proposed methods, we implement the generalized convolution operations as follows:

1. *Shift*: Auxiliary filter qubits and controlled quantum decrementers are used to create shifted (unity-strided) replicas of input data.
2. *Multiply-and-accumulate*: Arbitrary state synthesis and classical-to-quantum (C2Q) encoding are applied to create generic multidimensional filters.
3. *Data rearrangement*: Quantum permutation operations are applied to restructure the fragmented data into one contiguous output datum.

In Section 3.1, we present our quantum convolution technique in detail for one-dimensional data. In the following sections, we illustrate optimizations to improve circuit depth and generalize our method for multidimensional data. For evaluating our proposed methods, we used real-world, high-resolution, black-and-white (B/W) and RGB images, ranging in a resolution from (8×8) pixels to (512×512) pixels and (8×8×3) pixels to (512×512×3) pixels, respectively. We also performed experiments on 1-D real-world audio data and 3-D real-world hyperspectral data to demonstrate our method’s applicability to data and filters of any dimensionality. Further details about our experimental setup and dataset can be found in Section 4.

### 3.1. Quantum Convolution for One-Dimensional Data

**Figure 3:**
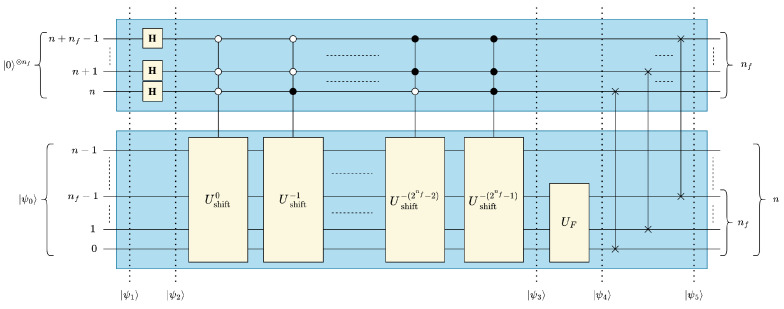
The 1-D quantum convolution circuit.

The proposed structure of quantum convolution for one-dimensional (1-D) data is shown in Figure 3. The following sections show the details of the five steps of the convolution operation procedure to transform the initial encoded data $|\psi_{0}\rangle$ to the final state $|\psi_{5}\rangle$, see Figure 3.

#### 3.1.1. Shift Operation

To perform convolution with unity stride with a filter of size $N_{f}$ terms, $N_{f}$ replicas of the input data must be made, strided for $0\leq k<N_{f}$. To store these replicas, we add $n_{f}=\left\lceil \log_{2}N_{f}\right\rceil$ auxiliary qubits, which we denote as “filter qubits”, to the most significant positions of the initial quantum state $|\psi_{0}\rangle$, see (4) and Figure 3:

(4) |ψ1〉=|0〉⊗nf⊗|ψ0〉=|ψ0〉0⋮0|ψ0〉2n0⋮02n+nf

Placing the filter qubits in superposition using Hadamard (H) gates creates $2^{n_{f}}$ identical replicas of the initial data $|\psi_{0}\rangle$, as shown in (5):

(5) |ψ2〉=H⊗nf⊗I⊗n|ψ1〉=12nf|ψ0〉⋮|ψ0〉|ψ0〉2n⋮|ψ0〉2n2n+nf

Finally, multiplexed quantum shift operations can be used to generate the strided replicas, see (6):

(6) |ψ3〉=Umux|ψ2〉=12nfUshift0|ψ0〉⋮Ushift−(2nf−1)|ψ0〉Ushift0|ψ0〉2n⋮Ushift−(2nf−1)|ψ0〉2n2n+nf,whereUmux=Ushift0⋱Ushift−(2nf−1)

#### 3.1.2. Multiply-and-Accumulate Operation

For the traditional convolution operation, applying a filter $F\in R^{N_{f}}$ to an array of data $W\in R^{N_{f}}$ produces a scalar output x∈R, which can be expressed as $x=F^{T}W$. In the quantum domain, we can represent an array of data as the partial state |ϕ〉 and the normalized filter as |F〉. Accordingly, the output state can be expressed as shown in (7):

(7) $$\begin{array}{cc} |\psi_{\mathrm{out}}\rangle & =\sum_{i=0}^{2^{n}-1}\langle F|\phi_{i}\rangle \cdot |i\rangle ,\;\mathrm{where}\;\\ |\phi_{i}\rangle & =\sum_{j=0}^{2^{n_{f}}-1}\langle k^{\prime }|\psi_{3}\rangle \cdot |k^{\prime }\rangle ,\;\mathrm{and}\;k^{\prime }=\left(2^{n_{f}}\cdot i\right)+j \end{array}$$

To calculate $|\psi_{4}\rangle$ from $|\psi_{3}\rangle$, it is necessary to embed 〈*F*| into a unitary operation $U_{F}$ as shown in (8). Since 〈*F*| is a normalized row vector, we can define $U_{F}$ as a matrix such that its first row is 〈*F*| and the remaining rows are arbitrarily determined to preserve the unitariness of $U_{F}$ such that $U_{F}^{†}U_{F}=U_{F}U_{F}^{†}=I^{⊗n_{f}}$. From (2), we can realize $U_{F}$ using an inverse C2Q operation, see (8):

(8) |ψ4〉=I⊗n⊗UF|ψ3〉=UF|ϕ0〉⋮UF|ϕ2n−1〉UF|ϕ0〉2nf⋮UF|ϕ2n〉−12nf2n+nf,whereUF=〈F|〈×|⋮〈×|2nf=UC2Q†

#### 3.1.3. Data Rearrangement

As of $|\psi_{4}\rangle$, the desired values of $|\psi_{\mathrm{out}}\rangle$ are fragmented among undesired/extraneous values, which we denote using the symbol “×”. We apply SWAP permutations to rearrange and coalesce our desired values to be contiguous in the final statevector $|\psi_{5}\rangle$, see (9) and Figure 3:

(9) |ψ5〉=USWAP1−D|ψ4〉=〈F|ϕ0〉⋮〈F|ϕ2n−1〉×⋮×Fϕ0⋮Fϕ2n−12n×⋮×2n+nf=|ψout〉×⋮×|ψout〉2n×⋮×2n+nf,whereUSWAP1−D=∏j=nf−10I⊗(nf−1−j)⊗SWAPj,j+n⊗I⊗j

#### 3.1.4. Circuit Depth Analysis of 1-D Quantum Convolution

When considering the circuit depth complexity of the proposed 1-D quantum convolution technique, it is evident from Figure 3 that the operations described by (5) and (9) are performed using parallel Hadamard and SWAP operations, respectively, and thus are of constant depth complexity, i.e., O(1). In contrast, the $U_{\mathrm{mux}}$ and $U_{F}$ operations incur the largest circuit depth, as they are both serial operations that scale with the data size and/or filter size, see Figure 3.

For the implementation of $U_{\mathrm{mux}}$ in Figure 3, there are a total of $2^{n_{f}}$ controlled quantum shift operations, where the *i*-th shift operation is a quantum decrementer $U_{\mathrm{shift}}^{-i}={\left(U_{\mathrm{shift}}^{-1}\right)}^{i}$. Since all the $U_{\mathrm{shift}}^{-1}$ operations are performed in series, the circuit depth of $U_{\mathrm{mux}}$ depends on the total number of unity quantum decrementers, $N_{U_{\mathrm{shift}}^{-1}}$, see (10):

(10) $$\begin{array}{cc} N_{U_{\mathrm{shift}}^{-1}}\left(n_{f}\right) & =\sum_{i=0}^{2^{n_{f}}-1}i=\frac{2^{n_{f}}\left(2^{n_{f}}-1\right)}{2}=\frac{4^{n_{f}}}{2}-2^{n_{f}-1} \end{array}$$

As shown in Figure 2b, each quantum decrementer $U_{\mathrm{shift}}^{-1}$ acting on *m* qubits can be realized using *m* multi-controlled CNOT (MCX) gates, where the *i*-th MCX gate is controlled by *i* qubits and 0≤i<m. Accordingly, for each quantum decrementer $U_{\mathrm{shift}}^{-1}$ that is controlled by *c* qubits, its *i*-th MCX gate is controlled by a total of i+c qubits. Therefore, the depth of the quantum decrementer circuits can be expressed in terms of the MCX gate count as shown in (11):

(11) $$\begin{array}{cc} D_{U_{\mathrm{shift}}^{-1}}\left(m,c\right) & =\sum_{i=c}^{m+c-1}D_{\mathrm{MCX}}\left(i\right) \end{array}$$

The depth of an MCX gate with a total of *m* qubits can be expressed with a linear function in terms of fundamental single-qubit rotation gates and CNOT gates [19] as shown in (12), where α represents the first-order coefficient and β represents the constant bias term. Thus, the depth complexity of $U_{\mathrm{shift}}^{-1}$ can be expressed as shown in (13):

(12) $$\begin{array}{cc} D_{\mathrm{MCX}}\left(m\right) & =\alpha m+\beta :\alpha ,\beta \in R \end{array}$$

(13) $$\begin{array}{cc} D_{U_{\mathrm{shift}}^{-1}}\left(m,c\right)\; & =\sum_{i=0}^{m-1}\alpha \left(i+c\right)+\beta \\ \; & =\frac{\alpha }{2}m^{2}+\left(\alpha \left(c-\frac{1}{2}\right)+\beta \right)m\\ \; & =O\left(m^{2}\right) \end{array}$$

To derive the circuit depth complexity of $U_{\mathrm{mux}}$, we leverage the definitions of $N_{U_{\mathrm{shift}}^{-1}}\left(n_{f}\right)$ and $D_{U_{\mathrm{shift}}^{-1}}\left(m,c\right)$ as shown in (14), where m=n and $c=n_{f}$:

(14) $$\begin{array}{cc} D_{U_{\mathrm{mux}}}\left(n,n_{f}\right) & =N_{U_{\mathrm{shift}}^{-1}}\left(n_{f}\right)\cdot D_{U_{\mathrm{shift}}^{-1}}\left(n,n_{f}\right)\\ \; & =\left(\frac{4^{n_{f}}}{2}-2^{n_{f}-1}\right)\cdot \left(\frac{\alpha }{2}n^{2}+\left(\alpha \left(n_{f}-\frac{1}{2}\right)+\beta \right)n\right)\\ \; & =\left(4^{n_{f}-1}-2^{n_{f}-2}\right)\cdot \left(\alpha n^{2}+2\alpha n_{f}n-\left(\alpha -2\beta \right)n\right)\\ \; & =O\left(4^{n_{f}}n^{2}+4^{n_{f}}n_{f}n\right) \end{array}$$

As discussed in Section 3.1.2, we implement the filter operation $U_{F}$ by leveraging the C2Q arbitrary synthesis operation [17]. Although C2Q incurs a circuit depth of exponential complexity in terms of fundamental quantum gates, as shown in (15), $U_{F}$ is only applied to $n_{f}$ qubits, a small subset of the total number of qubits, which somewhat mitigates the circuit depth. Furthermore, in most practical scenarios, the dimensions of the filter are typically much smaller than the dimensions of the input data, i.e., $n_{f}\ll n$. As a result, $U_{F}$ should not incur overly large circuit depth relative to other circuit components, e.g., $U_{\mathrm{mux}}$. Altogether, the overall circuit depth complexity of the 1-D quantum convolution operation can be expressed according to (16):

(15) $$D_{U_{F}}\left(n_{f}\right)=O\left(2^{n_{f}}\right)$$

(16) $$D_{1-D\;\mathrm{conv}}\left(n,n_{f}\right)=O\left(4^{n_{f}}n^{2}+4^{n_{f}}n_{f}n+2^{n_{f}}\right),\;\mathrm{where}\;n\gg n_{f}$$

### 3.2. Depth-Optimized 1-D Quantum Convolution

**Figure 4:**
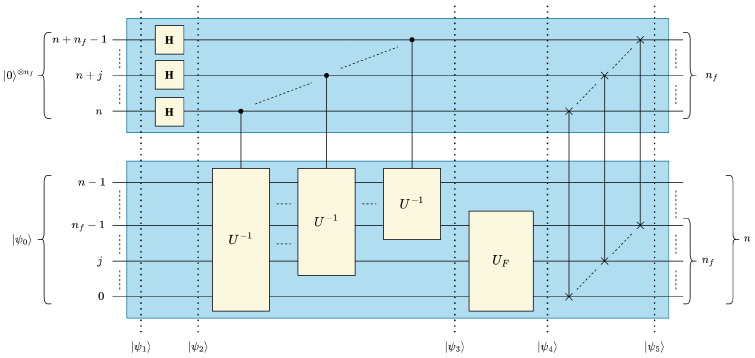
Depth-optimized 1-D quantum convolution circuit.

In Figure 4, we present an optimized implementation of $U_{\mathrm{mux}}$ that greatly reduces the circuit depth.

In Section 3.1, we implemented $U_{\mathrm{mux}}$ with $2^{n_{f}}$ controlled quantum decrementers $U_{\mathrm{shift}}^{-k}$, where $0\leq k<2^{n_{f}}$. We can represent each *k* in binary notation, as shown in (17), to express $U_{\mathrm{shift}}^{-k}$ as a sequence of controlled shift operations by powers of 2. As shown in (18), we can denote such operations with the notation $U_{\mathrm{shift}}^{-b_{j}2^{j}}\left(n\right)$, where $0\leq j<n_{f}$, and (n) reflects that the shift operation is applied to an *n*-qubit state.

(17) $$\begin{array}{cc} k & ={\left(b_{n_{f}-1}b_{n_{f}-2}\cdots b_{j}\cdots b_{1}b_{0}\right)}_{2}=\sum_{j=0}^{n_{f}-1}b_{j}2^{j}:b_{j}\in \left\{0,1\right\} \end{array}$$

(18) $$\begin{array}{cc} U_{\mathrm{shift}}^{-k}\left(n\right) & =U_{\mathrm{shift}}^{-\sum_{j=0}^{n_{f}-1}b_{j}2^{j}}\left(n\right)=\prod_{j=0}^{n_{f}-1}U_{\mathrm{shift}}^{-b_{j}2^{j}}\left(n\right) \end{array}$$

The binary decomposition of the uniformly controlled $U_{\mathrm{shift}}^{-k}$ operations is conducive to several circuit depth optimizations. As shown in (19), the value of $b_{j}$ is dependent only on the state of the *j*-th filter qubit $q_{n+j}$. In other words, each $U_{\mathrm{shift}}^{-2^{j}}\left(n\right)$ can only be controlled by one qubit $q_{n+j}$, independently from the other control qubits. Accordingly, it is possible to coalesce the multiplexed $U_{\mathrm{shift}}^{-2^{j}}\left(n\right)$ operations across the *k* control indices. The resultant implementation of $U_{\mathrm{mux}}$, therefore, becomes a sequence of $2^{n_{f}}$ single-controlled $U_{\mathrm{shift}}^{-2^{j}}\left(n\right)$ operations, where $0\leq j<n_{f}$, which comparatively has a smaller circuit depth by a factor of $2^{n_{f}}$. Furthermore, each $U_{\mathrm{shift}}^{-2^{j}}\left(n\right)$ operation can be implemented using a single $U_{\mathrm{shift}}^{-1}\left(n-j\right)$ operation in lieu of sequential $U_{\mathrm{shift}}^{-1}\left(n\right)$ operations, see (20) and Figure 4, further reducing the depth by a factor of $2^{j}$ per operation:

(19) $$b_{j}=\left\{\begin{array}{cc} 1, & |q_{n+j}\rangle =|1\rangle \\ 0, & \mathrm{otherwise} \end{array}\right.,\;\forall k\in \left[0,2^{n_{f}}\right]$$

(20) $$U_{\mathrm{shift}}^{-2^{j}}\left(n\right)\equiv \prod_{2^{j}}^{}U_{\mathrm{shift}}^{-1}\left(n\right)=U_{\mathrm{shift}}^{-1}\left(n-j\right)⊗I^{⊗j}$$

#### Circuit Depth Analysis of Optimized 1-D Quantum Convolution

With the aforementioned optimizations, the depth of the updated $U_{\mathrm{mux}}$ operation can be expressed in terms of $D_{U_{\mathrm{shift}}^{-1}}\left(m,c\right)$ as described by (21), where m=n−j and c=1 for all $0\leq j<n_{f}$. In comparison with the depth complexity of the unoptimized $U_{\mathrm{mux}}$, see (14), the dominant term remains quadratic, i.e., $n^{2}$, in terms of the data qubits *n*. However, its coefficient is improved exponentially, from $4^{n_{f}}$ to $n_{f}$, see (14) and (21). Note that a cubic term $n_{f}^{3}$ in terms of the number of filter qubits is introduced in the optimized $U_{\mathrm{mux}}$ implementation, see (21). However, when considering the total depth for the optimized 1-D quantum convolution circuit $D_{1-D\;\mathrm{conv}}^{\mathrm{opt}}$, the $n_{f}^{3}$ term becomes negligible because of $U_{F}$, whose complexity $O(2^{n_{f}})$ is exponential in terms of the number of filter qubits, see (15), (21), and (22):

(21) $$\begin{array}{cc} D_{U_{\mathrm{mux}}}^{\mathrm{opt}}\left(n,n_{f}\right) & =\sum_{j=0}^{n_{f}-1}D_{U_{\mathrm{shift}}^{-1}}\left(n-j,1\right)=\sum_{j=0}^{n_{f}-1}\left[\frac{\alpha }{2}{\left(n-j\right)}^{2}+\left(\frac{\alpha }{2}+\beta \right)\left(n-j\right)\right]\\ \; & =\frac{\alpha }{2}n_{f}n^{2}-\frac{\alpha }{2}n_{f}^{2}n+\left(\alpha +\beta \right)n_{f}n+\frac{\alpha }{6}n_{f}^{3}-\frac{\alpha +\beta }{2}n_{f}^{2}+\frac{2\alpha +3\beta }{6}n_{f}\\ \; & =O\left(n_{f}n^{2}-n_{f}^{2}n+n_{f}^{3}\right),\;\mathrm{where}\;n\gg n_{f} \end{array}$$

(22) $$\begin{array}{cc} D_{1-D\;\mathrm{conv}}^{\mathrm{opt}}\left(n,n_{f}\right)\; & =O\left(n_{f}n^{2}-n_{f}^{2}n+n_{f}^{3}+2^{n_{f}}\right)\\ \; & =O\left(n_{f}n^{2}-n_{f}^{2}n+2^{n_{f}}\right),\;\mathrm{where}\;n\gg n_{f} \end{array}$$

### 3.3. Generalized Quantum Convolution for Multidimensional Data and Filters

**Figure 5:**
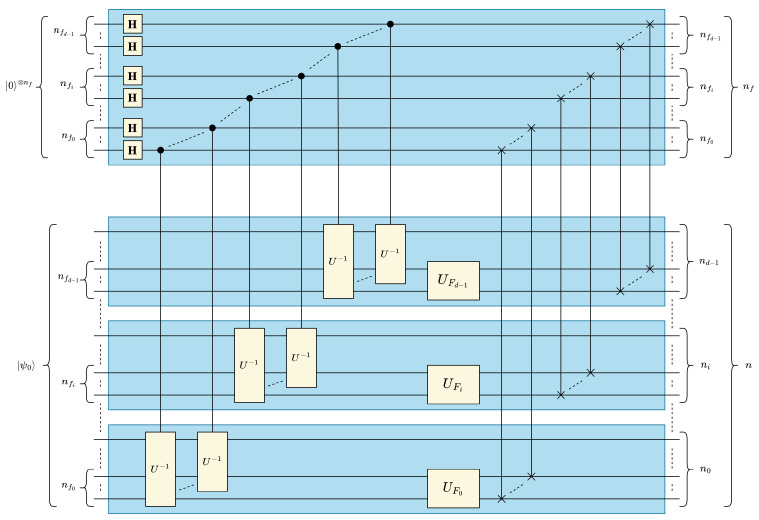
Multidimensional quantum convolution circuit with distributed/stacked 1-D filters.

In this section, we present the quantum circuit of our proposed quantum convolution technique generalized for multidimensional data and filters. Although quantum statevectors are one-dimensional, it is possible to map multidimensional data to a 1-D vector in either *row-* or *column*-major order. In this work, we represent multidimensional input data and convolutional filters in a quantum circuit in *column-major order*. In other words, for *d*-dimensional data of size $\left(N_{0}\times \cdots \times N_{i}\cdots \times N_{d-1}\right)$ data values, the positional information of the *i*-th dimension is encoded in the $\sum_{j=0}^{i-1}n_{j}$ to $(\sum_{j=0}^{i}n_{j})-1$ qubits, where $n_{i}=\left\lceil \log_{2}N_{i}\right\rceil$. Using this representation, the optimized 1-D quantum convolution circuit shown in Figure 4 can be generalized for *d* dimensions by *“stacking” d* 1-D circuits as shown in Figure 5.

**Figure 6:**
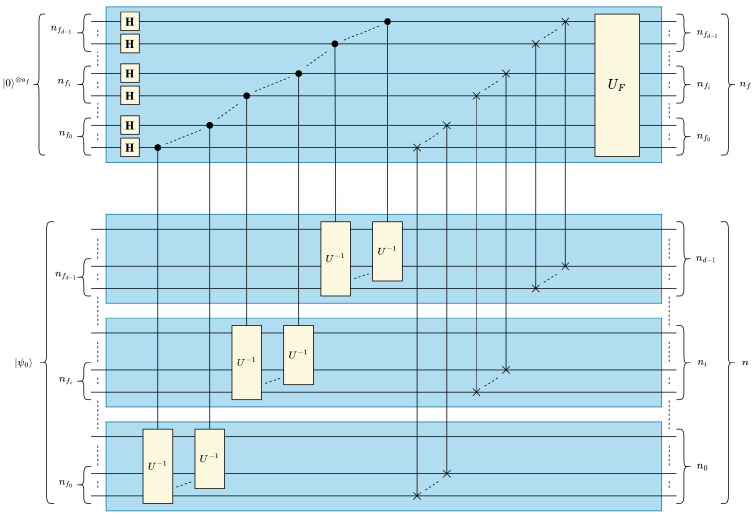
Generalized multidimensional quantum convolution circuit.

The “stacked” quantum circuit in Figure 5 is based on the assumption that the overall (lumped) *d*-dimensional filter operator $U_{F}$ is separable and decomposable into *d* one-dimensional filters $U_{F_{i}}$ for 0≤i<d. However, it would be more practically useful to generalize our multidimensional quantum convolution technique independently from the separability/decomposability of $U_{F}$. For this purpose, the identity in (23), which could be easily derived from either Figure 3 or Figure 4 for 1-D convolution, can be leveraged and generalized for multidimensional convolution circuits, see (24). The identity in (24) allows us to reverse the order of multiply-and-accumulate and data rearrangement steps and, therefore, generate one generic lumped $U_{F}$ that acts on the contiguous $n_{f}=\sum_{i=0}^{d-1}\left(n_{f_{i}}\right)$ filter qubits, where $n_{f_{i}}$ is the number of qubits representing the filter dimension *i* for 0≤i<d, see Figure 6. $U_{F}$ can be derived based on the given arbitrary multidimensional filter *F* using the method discussed in Section 3.1.2 when *F* is represented as a normalized 1-D vector |F〉 in a column major ordering:

(23) $$U_{\mathrm{SWAP}}^{1-D}\cdot \left(I^{⊗n}⊗U_{F}\right)=\left(U_{F}⊗I^{⊗n}\right)\cdot U_{\mathrm{SWAP}}^{1-D}$$

(24) $$\begin{array}{cc} U_{\mathrm{SWAP}}^{d-D} & \cdot \left(I^{⊗n_{f}}⊗\left(\overset{0}{\underset{i=d-1}{⨂}}\left[I^{⊗(n_{i}-n_{f_{i}})}⊗U_{F_{i}}\right]\right)\right)\begin{array}{cc} \; & \\ \; & =\left(\left(\overset{0}{\underset{i=d-1}{⨂}}\left[U_{F_{i}}\right]\right)⊗I^{⊗n}\right)\cdot U_{\mathrm{SWAP}}^{d-D}\\ \; & =\left(U_{F}⊗I^{⊗n}\right)\cdot U_{\mathrm{SWAP}}^{d-D},\;\mathrm{where} \end{array}\\ U_{F} & =\overset{0}{\underset{i=d-1}{⨂}}\left[U_{F_{i}}\right]\equiv U_{F_{d-1}}⊗U_{F_{d-2}}⊗\cdots ⊗U_{F_{1}}⊗U_{F_{0}},\\ U_{\mathrm{SWAP}}^{d-D} & =\prod_{i=d-1}^{0}\prod_{j=n_{f_{i}}-1}^{0}\left(I^{⊗\left(n_{f}-1-j-q_{f_{i}}\right)}⊗\mathrm{SWAP}\left(j+q_{i},j+n+q_{f_{i}}\right)⊗I^{⊗(j+q_{i})}\right),\\ q_{f_{i}} & =\sum_{k=0}^{i-1}n_{f_{k}},\;q_{i}=\sum_{k=0}^{i-1}n_{k},\;n_{f}=\sum_{k=0}^{d-1}n_{f_{k}},\;\mathrm{and}\;n=\sum_{k=0}^{d-1}n_{k} \end{array}$$

#### Circuit Depth Analysis of Generalized Multidimensional Quantum Convolution

As a result of the *“stacked"* structure, the data of all *d* dimensions could be concurrently processed in parallel. Consequently, the circuit depth of the multidimensional quantum circuit becomes dependent on the largest data dimension $N_{\max}=2^{n_{\max}}$, where $n_{\max}=\max_{i=0}^{d-1}\left(n_{i}\right)$, in lieu of the total data size *N*. The circuit component of the optimized 1-D circuit with the greatest depth contribution $U_{\mathrm{mux}}$ is performed in parallel on each dimension in the generic *d*-D circuit. Specifically, $U_{\mathrm{mux}}$ scales with the number of qubits used to represent the largest data dimension $n_{\max}=\left\lceil \log_{2}N_{\max}\right\rceil$. Note that the parallelization across dimensions applies to the Hadamard and SWAP operations from (5) and (9), see Figure 6, and therefore these operations are of constant depth complexity, i.e., O(1). The depth complexity of the multidimensional $U_{F}$ operation is determined by the total number of elements in the filter $N_{F}$, and therefore the C2Q-based implementation of $U_{F}$ does not benefit from multidimensional stacking. Accordingly, the circuit depth of the generalized multidimensional quantum convolution operation could be derived from (22) and expressed in (25), where $n_{f_{\max}}=\max_{i=0}^{d-1}\left(n_{f_{i}}\right)$ is the number of qubits representing the maximum filter dimension $N_{f_{\max}}=2^{n_{f_{\max}}}$. It is worth mentioning that the generic multidimensional formula in (25) reduces to the 1-D formula in (22) when $n=n_{\max}$ and $n_{f}=n_{f_{\max}}$:

(25) $$\begin{array}{cc} D_{d\text{-}D\;\mathrm{conv}}^{\mathrm{opt}}\left(n,n_{f}\right) & =O\left(n_{f_{\max}}n_{\max}^{2}-n_{f_{\max}}^{2}n_{\max}+2^{n_{f}}\right),\;\mathrm{where}\;\\ n_{\max} & =\max_{i=0}^{d-1}\left(n_{i}\right),\;n_{f_{\max}}=\max_{i=0}^{d-1}\left(n_{f_{i}}\right),\;\mathrm{and}\;n_{\max}\gg n_{f_{\max}} \end{array}$$

## 4. Experimental Setup and Results

**Figure 7:**
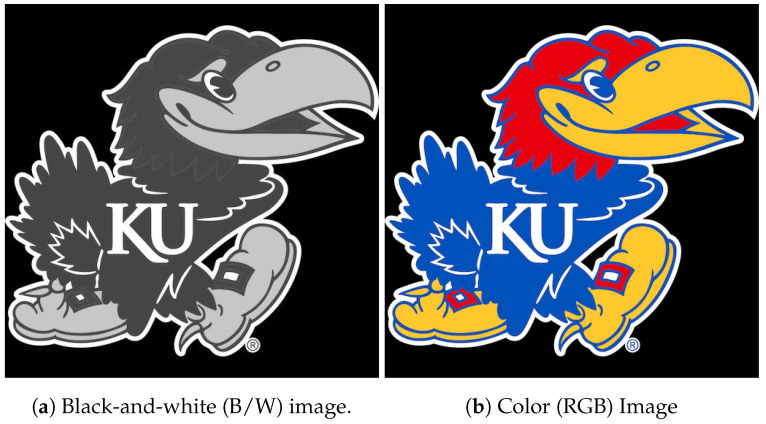
Real-world, high-resolution, multidimensional images used in experimental trials.

**Figure 8:**
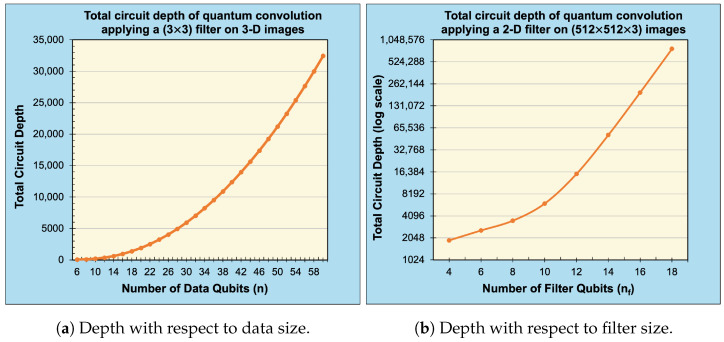
Circuit depth of quantum convolution with respect to data and filter qubits.

We experimentally demonstrate our proposed technique for generalized, multidimensional quantum convolution with unity stride on real-world, high-resolution, multidimensional image data, see Figure 7. By leveraging the Qiskit SDK (v0.39.4) from IBM Quantum [9], we simulate our quantum circuits in the following formats: (1) classically (to present the ideal/theoretical expectation), (2) noise-free (using statevector simulation), and (3) noisy (using 1,000,000 “shots” or circuit samples). Moreover, we present a quantitative comparison of the obtained results using fidelity [20] as a similarity metric between compared results ρ and σ, see (26). Experiments were performed on a 16-core AMD EPYC 7302P CPU with frequencies up to 3.3 GHz, 128 MB of L3 cache, and access to 256 GB of DDR4 RAM. In our analysis, we evaluated the correctness of the proposed techniques by comparing classical results with noise-free results. We also evaluated the scalability of the proposed techniques for higher-resolution images by comparing the classical results with both the noise-free and noisy results. Finally, we plotted the circuit depth of our techniques with respect to the data size and filter size as shown in Figure 8.

(26) $$\mathrm{Fidelity}\left(\rho ,\sigma \right)=\sqrt{\sqrt{\rho }\cdot \sigma \cdot \sqrt{\rho }}$$

In our experiments, we evaluated our techniques using well-known (3×3) and (5×5) filters, i.e., Averaging $F_{\mathrm{avg}}$, Sobel edge-detection $F_{\mathrm{Sx}}$/$F_{\mathrm{Sy}}$, Gaussian blur $F_{\mathrm{blur}}$, and Laplacian of Gaussian blur (Laplacian) $F_{L}$, see (27)–(30). We applied zero padding to maintain the size of the filter dimensions at a power of two for quantum implementation. In addition, we used wrapping to resolve the boundary conditions, and we restricted the magnitude of the output between [0,255] to mitigate quantization errors in the classical domain:

(27) $$\;\begin{array}{c} F_{\mathrm{avg}}^{3\times 3}=\frac{1}{9}\left[\begin{array}{ccc} 1 & 1 & 1\\ 1 & 1 & 1\\ 1 & 1 & 1 \end{array}\right], \end{array}\;\begin{array}{c} F_{\mathrm{avg}}^{5\times 5}=\frac{1}{25}\left[\begin{array}{ccccc} 1 & 1 & 1 & 1 & 1\\ 1 & 1 & 1 & 1 & 1\\ 1 & 1 & 1 & 1 & 1\\ 1 & 1 & 1 & 1 & 1\\ 1 & 1 & 1 & 1 & 1 \end{array}\right] \end{array}$$

(28) $$\begin{array}{c} F_{\mathrm{blur}}^{3\times 3}=\frac{1}{16}\left[\begin{array}{ccc} 1 & 2 & 1\\ 2 & 4 & 2\\ 1 & 2 & 1 \end{array}\right], \end{array}\;\begin{array}{c} F_{\mathrm{blur}}^{5\times 5}=\frac{1}{273}\left[\begin{array}{ccccc} 1 & 4 & 7 & 4 & 1\\ 4 & 16 & 26 & 16 & 4\\ 7 & 26 & 41 & 26 & 7\\ 4 & 16 & 26 & 16 & 4\\ 1 & 4 & 7 & 4 & 1 \end{array}\right] \end{array}$$

(29) $$\begin{array}{c} F_{\mathrm{Sx}}=\frac{1}{4}\left[\begin{array}{ccc} 1 & 0 & -1\\ 2 & 0 & -2\\ 1 & 0 & -1 \end{array}\right], \end{array}\;\begin{array}{c} F_{\mathrm{Sy}}=\frac{1}{4}\left[\begin{array}{ccc} 1 & 2 & 1\\ 0 & 0 & 0\\ -1 & -2 & -1 \end{array}\right] \end{array}$$

(30) $$\begin{array}{c} F_{L}^{3\times 3}=\frac{1}{6}\left[\begin{array}{ccc} 1 & 1 & 1\\ 1 & -8 & 1\\ 1 & 1 & 1 \end{array}\right], \end{array}\;\begin{array}{c} F_{L}^{5\times 5}=\frac{1}{20}\left[\begin{array}{ccccc} 1 & 1 & 1 & 1 & 1\\ 1 & 1 & 1 & 1 & 1\\ 1 & 1 & -24 & 1 & 1\\ 1 & 1 & 1 & 1 & 1\\ 1 & 1 & 1 & 1 & 1 \end{array}\right] \end{array}$$

**Figure 9:**
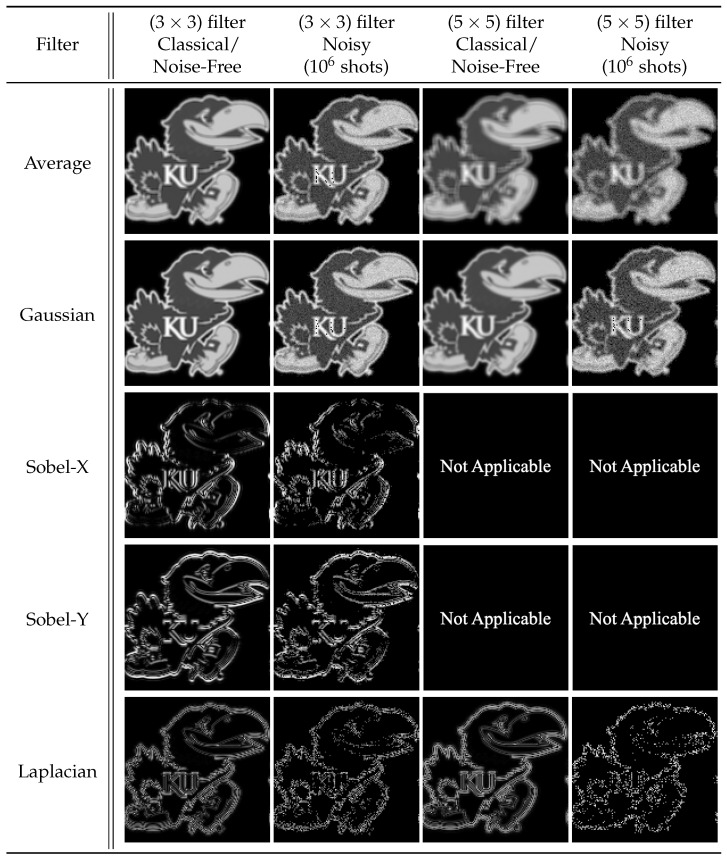
The 2-D convolution filters applied to a (128×128) B/W image.

**Figure 10:**
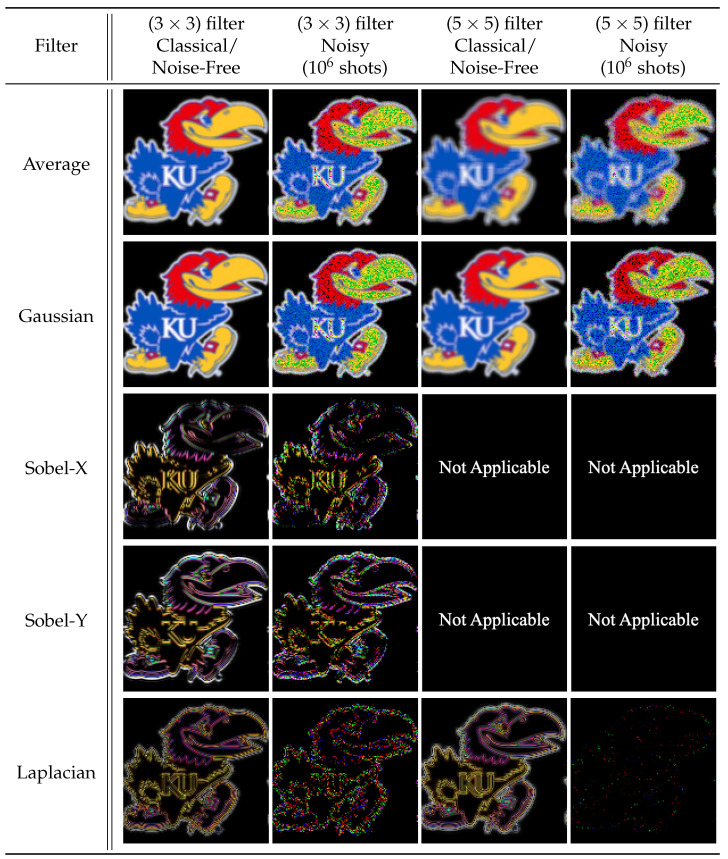
The 2-D convolution filters applied to a (128×128×3) RGB image.

We applied 2-D convolution filters to black-and-white (B/W) and RGB images, see Figure 7, ranging in resolution from (8×8) pixels to (512×512) pixels and (8×8×3) pixels to (512×512×3) pixels, respectively. The number of filter qubits can be obtained by the size of filter dimensions, i.e., $n_{f}=\left\lceil \log_{2}3\right\rceil +\left\lceil \log_{2}3\right\rceil =4$ qubits for (3×3) filters and $n_{f}=\left\lceil \log_{2}5\right\rceil +\left\lceil \log_{2}5\right\rceil =6$ qubits for (5×5) filters. Therefore, our simulated quantum circuits ranged in size ($n+n_{f}$) from 10 qubits to 26 qubits. Figure 9 and Figure 10 present the reconstructed output images for classical, noise-free, and noisy experiments using (128×128) and (128×128×3)-pixel input images, respectively.

**Figure 11:**
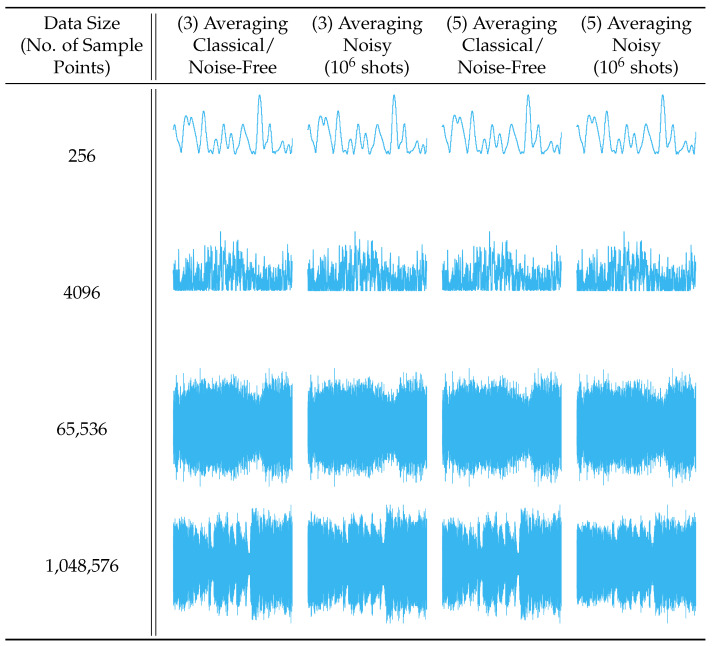
The 1-D convolution (averaging) filters applied to 1-D audio samples.

**Figure 12:**
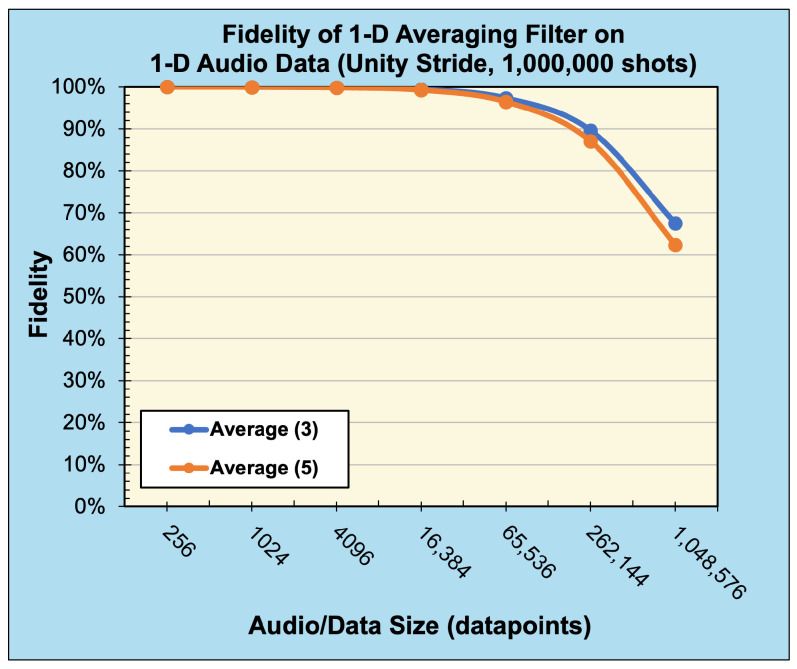
Fidelity of 1-D convolution (averaging) filters with unity stride on 1-D audio data (sampled with 1,000,000 shots).

**Figure 13:**
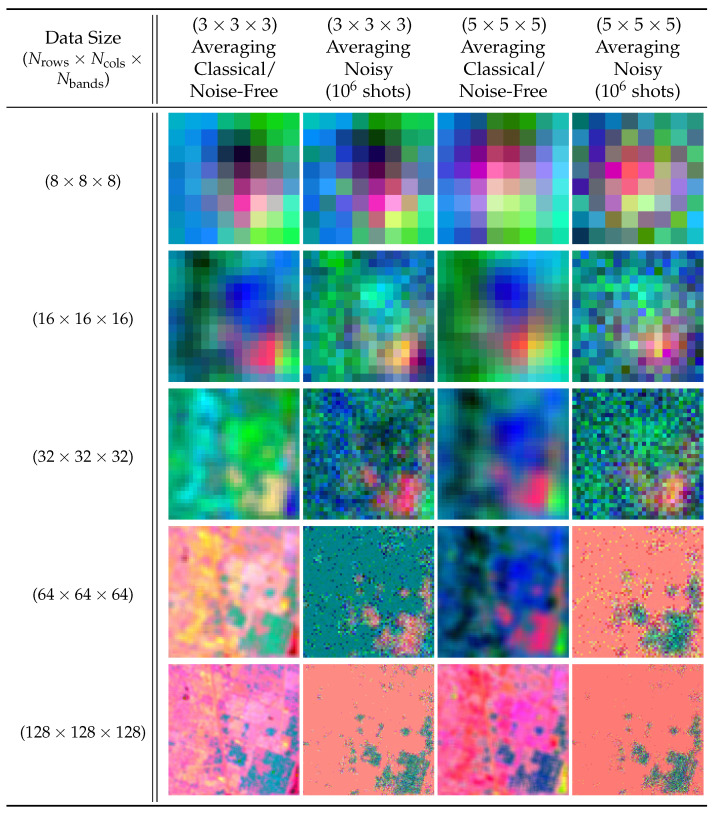
The 3-D convolution (averaging) filters applied to 3-D hyperspectral images (bands 0, 1, and 2).

**Figure 14:**
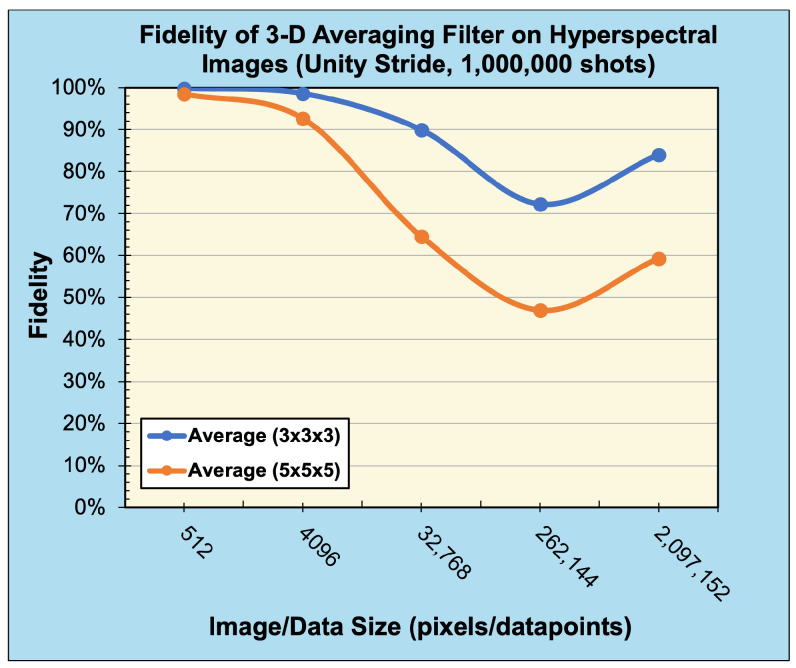
Fidelity of 3-D convolution (averaging) filters with unity stride on 3-D hyperspectral data (sampled with 1,000,000 shots).

To demonstrate our method’s applicability to data and filters of any dimensionality, we also performed experiments applying the 1-D and 3-D averaging filter to 1-D real-world audio data and 3-D real-world hyperspectral data, respectively. The audio files were sourced from the publicly available sound quality assessment material published by the European Broadcasting Union and modified to be single channel with data sizes ranging from $2^{8}$ values to $2^{20}$ values when sampled at 44.1 kHz [21]. Figure 11 and Figure 12 present the reconstructed output images and fidelity, respectively, from applying (3) and (5) averaging filters. The hyperspectral images were sourced from the Kennedy Space Center (KSC) dataset [22] and resized to range from (8×8×8) pixels to (128×128×128) pixels. Figure 13 and Figure 14 present the reconstructed output images and fidelity, respectively, from applying (3×3×3) and (5×5×5) averaging filters.

Comparison of the noise-free quantum results against the ideal classical results demonstrates a 100% fidelity across all trials. Thus, in a noise-free (statevector) environment, our proposed quantum convolution technique correctly performs an identical operation to classical convolution given the same input parameters and boundary conditions.

**Figure 15:**
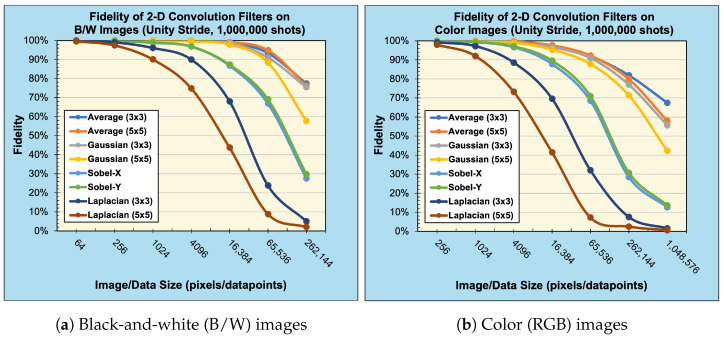
Fidelity of 2-D convolution with unity stride (sampled with 1,000,000 shots).

When considering the behavior of noisy (sampled) environments, Figure 12, Figure 14 and Figure 15 plot the fidelity of the noisy quantum results against the ideal classical results. We observe a monotonic decrease in fidelity as the data size (image resolution) increases, consistent with previously reported behavior [23]. Such behavior derives from how the number of shots required to properly characterize a quantum state increases with the corresponding number of qubits in order to reduce the effects of statistical noise. Notably, the fidelity varies dramatically depending on the filter category and size, where the largest discrepancy occurs between the (5×5) Averaging and (5×5) Laplacian filters for a data size of 65,536 values. Specifically, for a B/W image of (256×256) pixels, the Averaging filter had a fidelity of 94.84%, while the Laplacian filter had a fidelity of 8.82%—a difference of 86.02%, see Figure 15a. In general, we observed that the average and blur filters perform better than the edge detection methods (Sobel/Laplacian). Since the output data are reconstructed from only a portion of the final state $|\psi_{5}\rangle$, it is likely that sparse filters, represented in Figure 9 and Figure 10 as being mostly black, are significantly less likely to be recorded during sampled measurement, resulting in reduced fidelity. For practical applications, dimension reduction techniques, such as pooling, can be used to mitigate information loss [23].

## 5. Discussion

In the following section, we compare our proposed method of quantum convolution to the related work discussed in Section 2.1 in terms of filter generalization and circuit depth.

### 5.1. Arbitrary Multidimensional Filtering

Our generalizable and parameterized technique of quantum convolution with unity stride offers distinct workflow advantages over existing *fixed-filter* quantum convolution techniques in variational applications, such as quantum machine learning. Our technique does not require extensive development for each new filter design. For instance, current quantum convolutional filters are primarily two dimensional, only focusing on image processing. However, the development of even similar filters targeting audio and video processing, for example, would require extensive development and redesign. Our method offers a systematic and straightforward approach for generating practical quantum circuits given fundamental input variables.

### 5.2. Circuit Depth

Figure 8a,b show the circuit depth for our proposed technique of generalized quantum convolution with respect to the total number of data qubits $n=\sum_{i=0}^{d-1}\left(n_{i}\right)$ and the total number of filter qubits $n_{f}=\sum_{i=0}^{d-1}\left(n_{f_{i}}\right)$, respectively. The results were gathered using the QuantumCircuit.depth() method built into Qiskit for a QuantumCircuit transpiled to fundamental single-qubit and CNOT quantum gates. Figure 8a illustrates quadratic circuit depth complexity with respect to the data qubits *n* for a fixed filter size $N_{F}=2^{n_{f}}$, aligning with our theoretical expectation in (25). Note that $n=\sum_{i=0}^{d-1}n_{i}$ and $n_{\max}=\max_{i=0}^{d-1}\left(n_{i}\right)$ for *d*-dimensional data. Similarly, Figure 8b (plotted on a log-scale) illustrates exponential circuit depth complexity with respect to $n_{f}$ for a fixed data size $N=2^{n}$, which also aligns with our theoretical expectation in (25).

The time complexity comparison of our proposed quantum convolution technique against related work is shown in Table 1. Compared to classical direct implementations on CPUs, our proposed technique for generalized quantum convolution demonstrates an exponential improvement with respect to data size $N=2^{n}$, i.e., $O(n^{2})$ vs. $O(N^{2})$, see (25) and Table 1c. As discussed in Section 2.1, the fastest classical GEMM implementation of convolution on GPUs (excluding data I/O overhead) [12,13] has a complexity of $O(N_{F}N)$, see Table 1c. Even when including quantum data encoding, which is equivalent to data I/O overhead, our method remains to demonstrate a linear improvement with respect to data size *N* by a factor of the filter size $N_{F}$, see (31), over classical GEMM GPUs:

(31) $$\begin{array}{cc} D_{\mathrm{proposed}}\left(n\right) & =D_{C2Q}\left(n\right)+D_{d\text{-}D\;\mathrm{conv}}^{\mathrm{opt}}\left(n\right)\\ \; & =O\left(2^{n}+n_{\max}^{2}\right)=O\left(2^{n}\right)=O\left(N\right),\;\mathrm{for}\;\mathrm{fixed}\;n_{f} \end{array}$$

Compared to *fixed-filter* quantum convolution techniques [4,5,6,7,8], our proposed arbitrary filter quantum convolution technique (for unity stride) demonstrates improved circuit depth complexity with respect to data size when factoring in the circuit depth contribution from data encoding. For a fixed filter, i.e., $n_{f}$ is constant, the depth of the proposed method scales quadratically with the largest data dimension $n_{\max}$, see (25) and (31). As described in Section 2.1, *fixed-filter* quantum convolution techniques similarly show quadratic depth scaling with respect to the number of qubits *n*, see Table 1b. For data encoding, the *fixed-filter* techniques use either the FRQI [14] or NEQR [15] algorithms, which have circuit depth complexities of $O(4^{n})$ or $O(qn2^{n})$, respectively. In contrast, our proposed technique uses C2Q data encoding [17], which has a depth complexity of $O(2^{n})$—a quadratic and linear improvement over FRQI and NEQR, respectively, see Table 1a.

## 6. Conclusions

In this work, we proposed and evaluated a method for generalizing the convolution operation with arbitrary filtering and unity stride for multidimensional data in the quantum domain. We presented the corresponding circuits and their performance analyses along with experimental results that were collected using the IBM Qiskit development environment. In our experimental work, we validated the correctness of our method by comparing classical results to noise-free quantum results. We also demonstrated the practicality of our method for various convolution filters by evaluating the noisy quantum results. Furthermore, we presented experimentally verified analyses that highlight our technique’s advantages in terms of time complexity and/or circuit depth complexity compared to existing classical and quantum methods, respectively. Future work will focus on adapting our proposed technique for arbitrary strides. In addition, we will investigate multidimensional quantum machine learning as a potential application of our proposed technique.

## Figures and Tables

**Table 1:** Comparison of depth/time complexity of proposed generalized quantum convolution technique against related work.

**a** Depth complexity of quantum data encoding (I/O) techniques			
FRQI [14]	NEQR [15]	C2Q [17]	
$O_{I/O}\left(4^{n}\right)$	$O_{I/O}\left(qn2^{n}\right)$	$O_{I/O}\left(2^{n}\right)$	
**b** Depth complexity of quantum convolution algorithms for a fixed filter			
**Proposed**	Related Work [4,5,6,7,8]		
$O_{\mathrm{alg}}\left(n_{\max}^{2}\right)$	$O_{\mathrm{alg}}\left(n^{2}\right)$		
**c** Complexity of proposed technique compared to classical convolution			
**Proposed**	Direct (CPU) [10]	FFT (CPU/GPU) [10,11]	GEMM (GPU) [12,13]
$\begin{array}{c} O_{\mathrm{alg}}\left(\begin{array}{c} n_{f_{\max}}n_{\max}^{2}-n_{f_{\max}}^{2}n_{\max}\\ +2^{n_{f}} \end{array}\right)\\ O_{\mathrm{alg}}\;+\;I/O\left(\begin{array}{c} n_{f_{\max}}n_{\max}^{2}-n_{f_{\max}}^{2}n_{\max}\\ +2^{n}+2^{n_{f}} \end{array}\right) \end{array}$	$\begin{array}{c} O_{\mathrm{alg}}\left(N^{2}\right)\equiv \\ O_{\mathrm{alg}}\left(4^{n}\right) \end{array}$	$\begin{array}{c} O_{\mathrm{alg}}\left(N\log N\right)\equiv \\ O_{\mathrm{alg}}\left(n2^{n}\right) \end{array}$	$\begin{array}{c} O_{\mathrm{alg}}\left(N_{F}N\right)\equiv \\ O_{\mathrm{alg}}\left(2^{\left(n+n_{f}\right)}\right) \end{array}$

## Data Availability

The audio samples used in this work are publicly available from the European Broadcasting Union at https://tech.ebu.ch/publications/sqamcd (accessed on 19 October 2023) as file 64.flac [21]. The hyperspectral data used in this work are publicly available from the Grupo de Inteligencia Computacional (GIC) at https://www.ehu.eus/ccwintco/index.php/Hyperspectral_Remote_Sensing_Scenes#Kennedy_Space_Center_(KSC) (accessed on 19 October 2023) under the heading Kennedy Space Center (KSC) [22].

## Abbreviations

The following abbreviations are used in this manuscript:

1-D	One Dimensional
C2Q	Classical to Quantum
FFT	Fast Fourier Transform
FRQI	Flexible Representation of Quantum Images
GEMM	General Matrix Multiplication
NEQR	Novel Enhanced Quantum Representation
QHED	Quantum Hadamard Edge Detection
QWT	Quantum Wavelet Transform
